# 

**Published:** 1901-07-27

**Authors:** 


### NOTICE TO CORRESPONDENTS.

All MSS., Letters, Books for Review, and other matters intended for the Editor should be addressed The Editor, "The Hospital," The Hospital Building, 28 & 29 Southampton Street, Strand, W.C.

The Editor cannot undertake to return rejected MS., even when accompanied by stamped directed envelope.

**Notice to Advertisers and Subscribers.**

All Advertisements, Orders for Copies of The Hospital, and Business Communications should be addressed to **THE MANAGER (Not to the Editor),** The Hospital, 28 & 29 Southampton Street, Strand, London, W.C.

The Cost of Subscription, post free, is as follows:—Per week, 2½d.; per quarter, 2s. 8d.; per half-year, 5s. 3d.; per annum, 10s. 6d. Foreign:— Per annum, 15s. 2d., payable, in all cases, in advance.

**NOTICE.—Vol. XXIX. of "The Hospital" (October 1900, to March, 1901), in handsome cloth binding is now on sale at the Office of this Journal, price, 7s. 6d. Cases for binding the Numbers contained in this Volume 1s. 6d. Index to Vol. XXIX., 2½d., post free.**

**The Monthly Part for August will be ready on August 1st. Price 6d., by post 8d.**

### SCRAPS AND GLEANINGS.

THE Apollinaris Company, Limited, have been appointed by Royal warrant purveyors of natural mineral waters to His Majesty the King.

SOME necessary alterations and improvements in the out-patient department are contemplated at no very distant date by the committee of the Birmingham and Midland Eye Hospital.

AT the recent dinner of the London Association of Correctors of the Press, held under the presidency of Mr. Sheriff Lawrence, M.P., the sum of £300 was raised, which is a record collection for this association at their dinners.

THE average stay of each in-patient of the Gloucester Infirmary and Eye Institution during the past year was 27·6 days, which is slightly less than the average for the previous year. The number of patients treated during the year is 1,306.

THE bread sent in for competition by the leading bakers in the Midlands to the "Hovis" Bread Company was, after being judged, divided amongst St. Thomas's, the London, and Metropolitan Hospitals, Nazareth House, and Dr. Barnado's Home, by the inmates of all of which institutions it was greatly appreciated.

THE "Frame Food" Company are issuing a charming little booklet entitled "Our Boy and his Dog." The illustrations are so delightful that we advise those who wish to spread joy in the children's ward, or in the sick room of small patients, to take advantage of the offer of the company to supply it free of cost to anyone asking for it.

MR. F. W. SPEAIGHT, of Regent Street, has published a Souvenir Guide to the Children's Fête held at the Royal Botanical Gardens in aid of the National Society for the Prevention of Cruelty to Children. This book contains some very pretty photographs of the Committee and others taking part in the fête, and the proceeds of its sale are to be handed to the Countess of Ancaster towards the profits of the fête.

IT is expected that the women's ward at the Cardiff Infirmary will be opened in August. It will start with ten or eleven beds, and it is hoped that before long it may be possible to open the remaining five or six beds that the ward contains. £900 has already been offered for the half endowment and maintenance of a bed, on condition that within a year a similar sum is raised to complete the endowment, and if this condition is fulfilled it is probable that next year the same sum will be given towards another bed.

### THE STUDENT OF MEDICINE.

### APPOINTMENTS.

**W. Astin**, M.B., C.M.Aberd., appointed Certifying Factory Surgeon for the Loughton District of Essex. **G. C. R. Bull**, M.B.Lond., F.R.C.S.Eng., appointed Medical Officer for the Turvey District of the Bedford Union. **L. Vernon Cargill**, F.R.C.S., appointed Surgeon to the Royal Eye Hospital, Southwark. **E. E. Crawshaw**, M.R.C.S., L.R.C.P., appointed Resident Obstetric Officer to the General Infirmary, Leeds. **Harold Collinson**, M.B.Lond., M.R.C.S., L.R.C.P., appointed Resident Casualty Officer to the General Infirmary, Leeds. **H. F. Ealand**, L.R.C.P., L.R.C.S.Edin., appointed District Medical Officer to the Farnham Union. **G. Elliott**, L.R.C.P., L.R.C.S.Edin., appointed District and Workhouse Medical Officer of the Stamford Union. **P. J. Fagan**, F.R.C.S.I., appointed Lecturer on Elementary Physics in the Catholic University Medical School, Dublin. **C. Farrant**, M.R.C.S.Eng., L.R.C.P.Lond., appointed District Medical Officer of the Taunton Union. **John Wainman Findlay**, M.D., appointed Dispensary (or Assistant) Physician to the Glasgow Royal Infirmary. **R. T. Forster**, L.S.A., appointed Medical Officer for the Third District of the Bridlington Union. **Lewis D. Heather**, M.R.C.S., L.R.C.P.Lond., appointed District Medical Officer of the Hay Union. **A. E.**

Horn, M.B.Lond., appointed First Assistant Medical Officer of the Chelsea Infirmary. **James Brierley Hughes,** M.R.C.S.Eng., L.S.A., appointed Consulting Medical Officer to the Macclesfield General Infirmary. **John Brierley Hughes,** B.A., M.B., B.C.Cantab., M.R.C.S.Eng., L.R.C.P.Lond., appointed Honorary Member of Medical Staff of the Macclesfield General Infirmary. **J. W. Laver,** M.R.C.S., L.R.C.P.Lond., appointed Medical Officer for the Grimston Division of the Freebridge Lynn Union. **S. E. Martin,** M.D., M.Ch.R.U.I., appointed Certifying Factory Surgeon for the Newry District. **C. C. W. Mayo,** M.R.C.S., L.R.C.P.Lond., appointed Resident Assistant Medical Officer to the Ecclesall Bierlow Union Workhouse. **James Muirhead,** M.B., B.S.Durh., appointed Senior House-Physician to the Royal Infirmary, Newcastle-on-Tyne. **John Norton,** M.D.Durh., M.R.C.S.Eng., D.P.H.Eng., appointed Surgeon to the A Division Metropolitan Police (Great Scotland Yard). **T. O'Connell,** L.R.C.S.I., L.A.H.Dub., appointed Certifying Factory Surgeon for the Fethard and Tullamain Dispensary Districts, co. Tipperary. **T. E. Pallett,** M.D.Brux., L.S.A., appointed Medical Officer for the Tenth District of the Lexden and Winstree Union. **P. J. Probyn-Williams,** M.D., appointed Lecturer and Instructor in Anæsthetics at the London Hospital. **T. W. Robinson,** M.D., Ch.M., appointed District Medical Officer at Albany, Western Australia, and Vaccinator for the District of Albany. **Edmund R. Roseby,** M.B., appointed Resident Medical Officer at the Adelaide Hospital, South Australia. **Amand Routh,** M.D., B.S., F.R.C.P.Lond., appointed Consulting Physician to the Samaritan Free Hospital for Women, London. **G. J. Silver,** M.B., C.M.Aberd., appointed District Medical Officer of the Walsall Union. **R. B. Simmons,** M.B., appointed Medical Officer at Clermont, Queensland. **W. Simpson,** M.B., B.S.Durh., appointed Medical Officer for the Seventh District of the Newcastle-upon-Tyne Union. **Thomas Stang,** L.R.C.P., appointed Health Officer for the Shire of Echuca, Victoria. **E. J. Steegmann,** M.B.Durh., D.P.H.Eng., appointed Medical Officer of Health for the Heston and Isleworth Urban District. **John Steell,** M.B., Ch.M., appointed Medical Superintendent of the Ballarat Lunatic Asylum. **J. A. Swindale,** M.B., B.S.Durh., appointed Medical Officer for the Binfield District of the Easthampstead Union. **Thos. H. A. Valentine,** M.R.C.S.Eng., D.P.H., appointed Health Officer for Wellington District, New Zealand. **A. S. Vallack,** M.B., Ch.M., appointed Medical Officer and Vaccinator at Bowral. **Charles Wade,** M.R.C.S.Eng., L.R.C.P.Lond., appointed District Medical Officer of the Boscastle District of the Camelford Union.

